# *Nedl1* knockout ameliorates cognitive impairment and improves epilepsy threshold in pilocarpine-induced epileptic mice

**DOI:** 10.1186/s42494-024-00186-z

**Published:** 2025-01-13

**Authors:** Qian Lu, Mengjia Liu, Shufang Guo, Yangyang Wang, Liping Zou

**Affiliations:** 1 Department of Pediatrics, The First Medical Center, Chinese PLA General Hospital, Beijing, 100853 China; 2 Department of Pediatrics, First Hospital of Qinhuangdao, Hebei, 066000 China; 3 Beijing Children’s Hospital, Capital Medical University, Beijing, 100045 China; 4 Department of Pediatrics, General Hospital of Air Force, Beijing, 100048 China

**Keywords:** Epilepsy, Cognitive impairment, Knockout, *Nedl1*

## Abstract

**Background:**

Epilepsy is a common neurological disorder. The homologous to E6-AP carboxy terminus (HECT) E3 ligase is associated with epilepsy. NEDD4-like ubiquitin protein ligase-1 (NEDL1) is a HECT E3 ligase that is highly expressed in the brain. This study aimed to investigate the involvement of NEDL1 in epilepsy and the potential effect of NEDL1 on the cognitive ability.

**Methods:**

The pilocarpine-induced epileptic mouse model was used to assess cognitive functions in Barnes maze, the pathological changes, and the activation of astrocytes and microglia in wild-type (*Nedl1*^+*/*+^) and *Nedl1* knockout (*Nedl1*^*−/−*^) mice. The RNA-seq method was used to analyze differentially expressed genes and explore the brain pathophysiology after epilepsy development.

**Results:**

*Nedl1* knockout resulted in a protective effect against epilepsy. The *Nedl1*^*−/−*^ mice showed improved spatial learning and memory, alleviation of pathological damage in the hippocampus induced by epilepsy, and reduced microglial activation in the hippocampus. Kyoto Encyclopedia of Genes and Genomes (KEGG) analysis of differentially expressed genes also revealed several prominently enriched T-cell-related pathways.

**Conclusions:**

*Nedl1* knockout reduces seizures and alleviates neuroinflammation. The potential functional link between NEDL1 and epilepsy provides a new approach to the treatment and intervention of epilepsy.

**Supplementary Information:**

The online version contains supplementary material available at 10.1186/s42494-024-00186-z.

## Background

Epilepsy is a common neurological disease that affects individuals of all ages independent of ethnicity and geography. Approximately 4.9 million people in the world develop new-onset epilepsy annually [1]. Recurrent seizures not only cause harm to the patients but also burden their families and the health systems [2].

Ubiquitination is a common post-translational modification of proteins. Ubiquitin ligases mediate the binding of ubiquitin molecules to specific substrates. Based on different domains, ubiquitin ligases are classified into four categories: RING (really interesting new gene), HECT (homologous to the E6-AP carboxyl terminus), U-Box, and RBR (RING-in-between-RING) [3]. NEDD4-like ubiquitin protein ligase-1 (NEDL1) and NEDL2 are HECT ubiquitin ligases with similar sequences [3]. *NEDL2* variants have been found to be associated with epilepsy [4]. Our previous study also found that individuals with *NEDL2* variants had seizures [5]. Several HECT ubiquitin ligases have been reported to be associated with epilepsy. For example, loss of the UBE3A protein causes Angelman syndrome, which is characterized by seizures and developmental delay [6]. *Nedd4-2* haploinsufficiency in mice increases seizure susceptibility [7]. *C. elegans eel-1* mutants have decreased inhibitory GABAergic neuron function and increased seizure susceptibility [8]. A patient with a novel *HERC2* variant had refractory seizures and developmental delay [9].

Currently, the functions of NEDL1 in epilepsy remain poorly understood. In this study, we set out to explore the role of NEDL1 in epilepsy using *Nedl1*-knockout (*Nedl1*^*−/−*^) mice.

## Methods

### Animals

The first *Nedl1*^*−/−*^ mouse was gifted by the National Center of Protein Sciences (Beijing). The generation and genetic background of *Nedl1*^*−/−*^ mice have been described previously [10]. The established mice had systemic knockout of *Nedl1 *derived from the C57BL/6 strain. All experiments were performed in accordance with the guidelines approved by the Institutional Animal Care and Use Committee of Chinese PLA General Hospital (2013-X8-46).

Genotypes were confirmed by PCR from mouse tail DNA. The PCR primers for *Nedl1*^+*/*+^ were 5′-GTGCTGGAAATTGAAGTGAAGGACAA-3′ (forward) and 5′-ACAAACTACACAAGTATAAGAAGGGG-3′ (reverse). The PCR primers for *Nedl1*^−/−^ were 5′-CGCTACCATTACCAGTTGGTCT-3′ (forward) and 5′-TCGTATGGAAGTGCAGTATG-3′ (reverse). All animals were kept in specific-pathogen-free housing at a controlled temperature of 23 ± 2 °C with a humidity of 40–70% under a 12 h light-dark cycle. Feed, water, and bedding were autoclaved. Free diet and water harvesting were implemented. The bedding was changed twice a week.

### Pilocarpine-induced epileptic mouse model

*Nedl1*^+*/*+^ and *Nedl1*^*−/−*^ mice of both sexes at 2 months of age (weighing 20–30 g) were administered with 2 mg/kg methyl-scopolamine intraperitoneally (i.p.; Sigma-Aldrich, St. Luis, MO, USA). After 30 min, the mice received pilocarpine (245 mg/kg, i.p.; Sigma-Aldrich, St. Luis, MO, USA) to induce status epilepticus (SE). The aim of methyl-scopolamine administration was to reduce the peripheral cholinergic effects of pilocarpine. Behavioral changes of mice were video-recorded within 2 h after pilocarpine injection. Scoring was based on the Racine scale [11]. A score of stage 3 or higher indicated successful induction of SE [12]. SE was terminated with chloral hydrate (2 ml/kg, i.p.) after 2 h, which was produced by the PLA General Hospital.

### Whole-cell electrophysiology

Hippocampal slices (300-μm thick, *n* = 8 slices for each group) were prepared from *Nedl1*^+*/*+^ and *Nedl1*^*−/−*^ mice without pilocarpine treatment. After sectioning, the slices were incubated for at least 1 h in artificial cerebral spinal fluid containing (in mM): 124 NaCl, 2.5 KCl, 1.5 MgSO_4_, 1.2 NaH_2_PO_4_, 24 NaHCO_3_, 12.5 D-glucose, and 2 CaCl_2_ saturated with 95% O_2_ and 5% CO_2_ at pH 7.3. Sections in the pilocarpine group were incubated with 200 μM pilocarpine for 3 h. Pyramidal cells in the CA1 region of the hippocampus were identified by microscopy (Olympos BX50WI). The electrode filling solution contained (in mM) 140 K-gluconate, 2 MgCl_2_, 8 KCl, 10 HEPES, 0.2 NaGTP, and 2 Na_2_ATP. The number of action potentials, amplitude, and membrane potential within 5 min were recorded using the whole-cell patch-clamp technique.

### Barnes maze test

*Nedl1*^+*/*+^ and *Nedl1*^*−/−*^ mice underwent the Barnes maze test 1 month after SE. The Barnes maze was a white circular table placed 120 cm above the floor, with 20 holes at the outer border. The test was conducted 4 times a day with 15-min intervals, for 4 consecutive days. The mice were brought to the experimental site and placed in an opaque cup in the middle of the maze for 30 s before the experiment. Then, the cup was removed, and the mouse was allowed to explore the maze for 3 min. The test ended after 3 min or when the mouse entered the escape box. After the experiment, the mouse was placed into the escape box for 1 min. The latency to enter the escape box and the number of errors were recorded using a video tracking system.

### Hematoxylin and eosin (H&E) staining

*Nedl1*^*−/−*^ and *Nedl1*^+*/*+^ mice (*n* = 3 per group) were anesthetized 1 month after SE and transcardially perfused with 0.9% NaCl, followed by 4% paraformaldehyde. The brains were immediately removed and fixed overnight in 4% paraformaldehyde at 4 °C. The tissues were embedded in paraffin and sectioned at 5-μm thickness using a paraffin microtome. H&E staining was performed on these sections. Photographs were taken from regions of hippocampal CA1, CA3, and dentate gyrus (DG). The number of cells in each section was identified as the average number in three views of regions from the hippocampus.

### Immunohistochemistry

Brain sections (*n* = 3 per group) were deparaffinized and rehydrated, and underwent microwave antigen retrieval with sodium citrate. The sections were treated with normal serum in 0.01 M PBS for 30 min. Then, the sections were washed with PBS and incubated with rabbit anti-GFAP (Ab7260, Abcam, Cambridge, UK) and anti-Iba-1 (GB153502, Servicebio, Wuhan, China) overnight at 4 °C. Subsequently, the sections were incubated with biotinylated goat anti-mouse IgG and visualized with diaminobenzidine tetrahydrochloride. GFAP and Iba-1 immunofluorescence in hippocampal CA1, CA3, and DG regions was analyzed using the Image-Pro Plus 6.0 software.

### RNA sequencing

Total RNA of the brain was extracted from *Nedl1*^+*/*+^ and *Nedl1*^*−/−*^ mice (*n* = 3 per group). The total RNA from each sample was qualified and quantified using the Agilent 2100 bioanalyzer. One microgram of RNA with an RNA integrity number > 8 was used to prepare the library. A poly(A) mRNA magnetic isolation module was used to isolate poly(A) mRNA.

Samples were sequenced by Illumina HiSeq and the results were stored as FASTQ file. The sequencing results were compared with the reference genome by Tophat (v2.0.9) [13]. Fragments per kilobase of transcript per million mapped reads (FPKM) values were used to compare gene expression levels. Based on FPKM, principal component analysis was performed on genes to understand the correlation between different samples and the expression of genes. Differentially expressed genes (DEGs) between the two groups were analyzed using the DESeq R software. Genes with an adjusted *P* value < 0.05 were defined as DEGs. The DEGs underwent gene ontology (GO) and Kyoto Encyclopedia of Genes and Genomes (KEGG) pathway enrichment analyses.

### Statistical analysis

Data normal distribution was assessed using the Shapiro–Wilk test. For data without normal distribution, the nonparametric Mann–Whitney *U* test was used, otherwise the Student’s *t-*test was performed. Data from the Barnes maze test was not normally distributed, and they underwent repeated measures analysis of variance with the Scheierer-Ray-Hare test (companion R packags). *P* < 0.05 was considered as statistically significant. The data were analyzed using SPSS 22.0 (IBM, Armonk, NY), RStudio software (Posit, Boston, MA), and GraphPad Prism 7 software (GraphPad Software, Boston, MA).

## Results

### *Nedl1 *knockout reduces seizure severity and mortality in the pilocarpine model

**Fig. 1:**
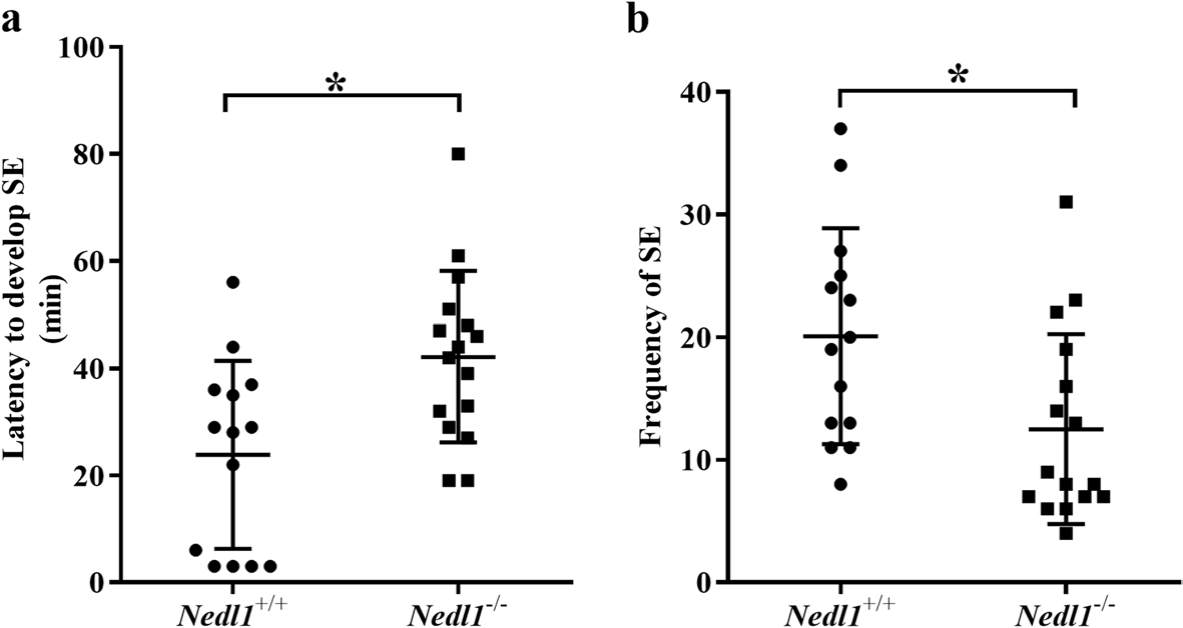
*Nedl1*-knockout mice show reduced seizure severity in the pilocarpine model. **a** The latency for seizure onset. **b** Seizure frequency within 2 h after administration of pilocarpine. *P < 0.05. Each dot represents data from an individual *Nedl1*^+*/*+^ mouse (*n* = 14) and each square represents data from an individual *Nedl1*.^*−/−*^ mouse (*n* = 16)

No morphological (hair, weight, appearance, etc.) difference was observed between *Nedl1*^*−/−*^ and *Nedl1*^+*/*+^ mice until 18 months of age [10]. No spontaneous seizures were found in *Nedl1*^*−/−*^ mice. *Nedl1*^*−/−*^ mice had a longer latency for seizure onset (42.13 ± 15.99 s) than *Nedl1*^+*/*+^ mice (23.86 ± 17.58 s, *P* = 0.012). The seizure frequency in *Nedl1*^*−/−*^ mice (12.50 ± 7.75 s) was less than that in *Nedl1*^+*/*+^ mice (20.07 ± 8.79 s, *P* = 0.012). Within 2 h of video monitoring, 11 *Nedl1*^+*/*+^ mice (total *n* = 25) died, with a mortality rate of 44%. None of the *Nedl1*^*−/−*^ mice died (*n* = 16, Fig. 1). Video of a representative *Nedl1*^+/+^ mouse that developed seizures a few minutes after pilocarpine administration is shown in Additional file 1: Video S1.

### Electrical activity of *Nedl1*^-/- ^mice changed slightly after pilocarpine injection

**Fig. 2:**
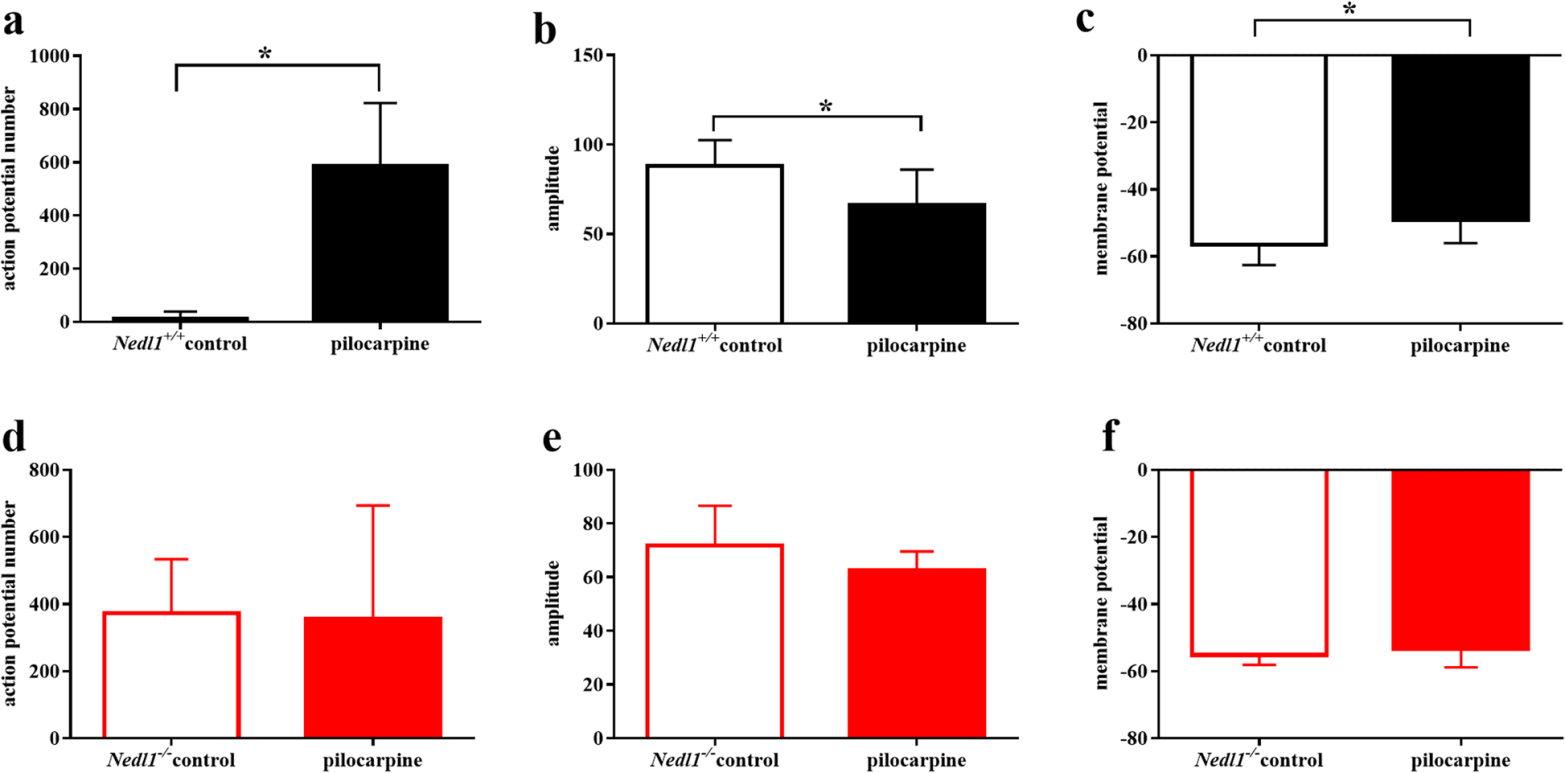
The electrical activity of *Nedl1*^*−/−*^ mice changed slightly after injection of pilocarpine. **a–c** show the number and the amplitude of action potentials*,* as well as the membrane potential of *Nedl1*^+*/*+^ mice. **d–f** show the number and the amplitude of action potentials*,* as well as the membrane potential of *Nedl1*^*−/−*^ mice. ^*^P < 0.05, *n* = 8 for each group

After injection of pilocarpine, the *Nedl1*^+*/*+^ mice showed significantly higher number of spontaneous action potentials (*P* = 0.001), lower amplitude of action potentials (*P* = 0.024), and increased membrane potential (*P* = 0.039) compared to the control group. However, the *Nedl1*^*−/−*^ mice showed no significant difference in the number of spontaneous action potentials, the amplitude of action potentials, and the membrane potential compared with the control group (*P* = 0.674, 0.137, and 0.416, respectively; Fig. 2).

### *Nedl1* knockout alleviates learning and memory impairment caused by epilepsy

**Fig. 3:**
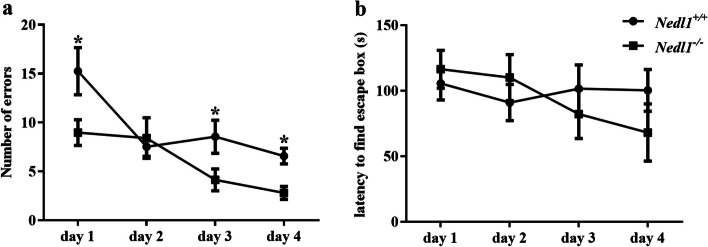
Barnes maze test results. *Nedl1* knockout ameliorated the learning and memory impairment induced by epilepsy. **a** Number of errors in finding the escape box. **b** The latency to find the escape box. ^*^P < 0.05

In the Barnes maze test, the two groups of mice showed a gradual decrease in the number of errors in finding an escape box. Results of Scheirer-Ray-Hare analysis showed statistical significance in the number of errors between groups and among test days, but no significant interaction effect between group and time (Additional file 2: Table S1). The number of errors of *Nedl1*^*−/−*^ mice was lower than that of *Nedl1*^+*/*+^ mice on day 1, day 3, and day 4 (*P* = 0.041, 0.048, and 0.020, respectively). There was no significant difference in the latency to find the escape box between the two groups (*P* > 0.05; Fig. 3 and Table S2).

### *Nedl1* knockout ameliorates neuronal cell damage and microglial activation in the *hippocampus* after epilepsy

**Fig. 4:**
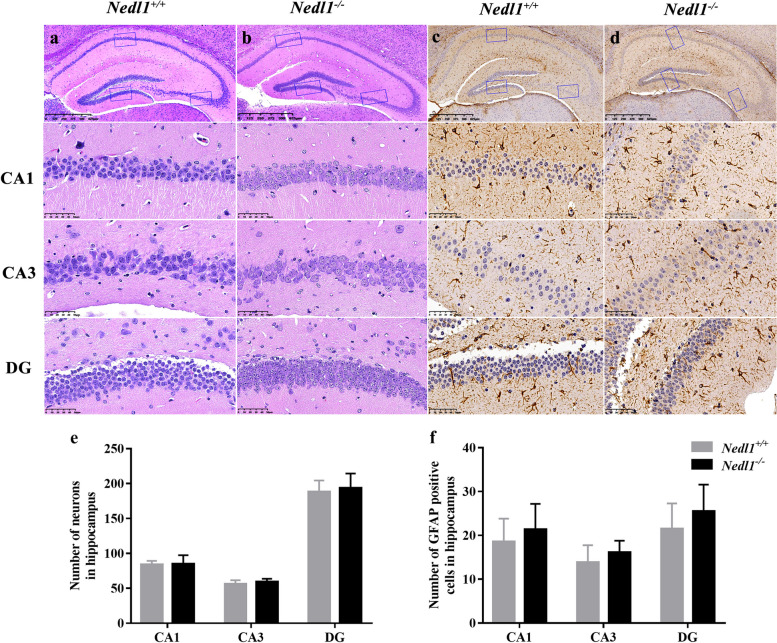
Neuronal and astrocytic staining in the hippocampus of mice after epilepsy. **a, b** H&E staining; **c, d** GFAP staining in the CA1, CA3 and DG region. **e** Quantitation of the number of neurons in the hippocampus. **f** Quantitation of the number of GFAP-positive cells in the hippocampus

**Fig. 5:**
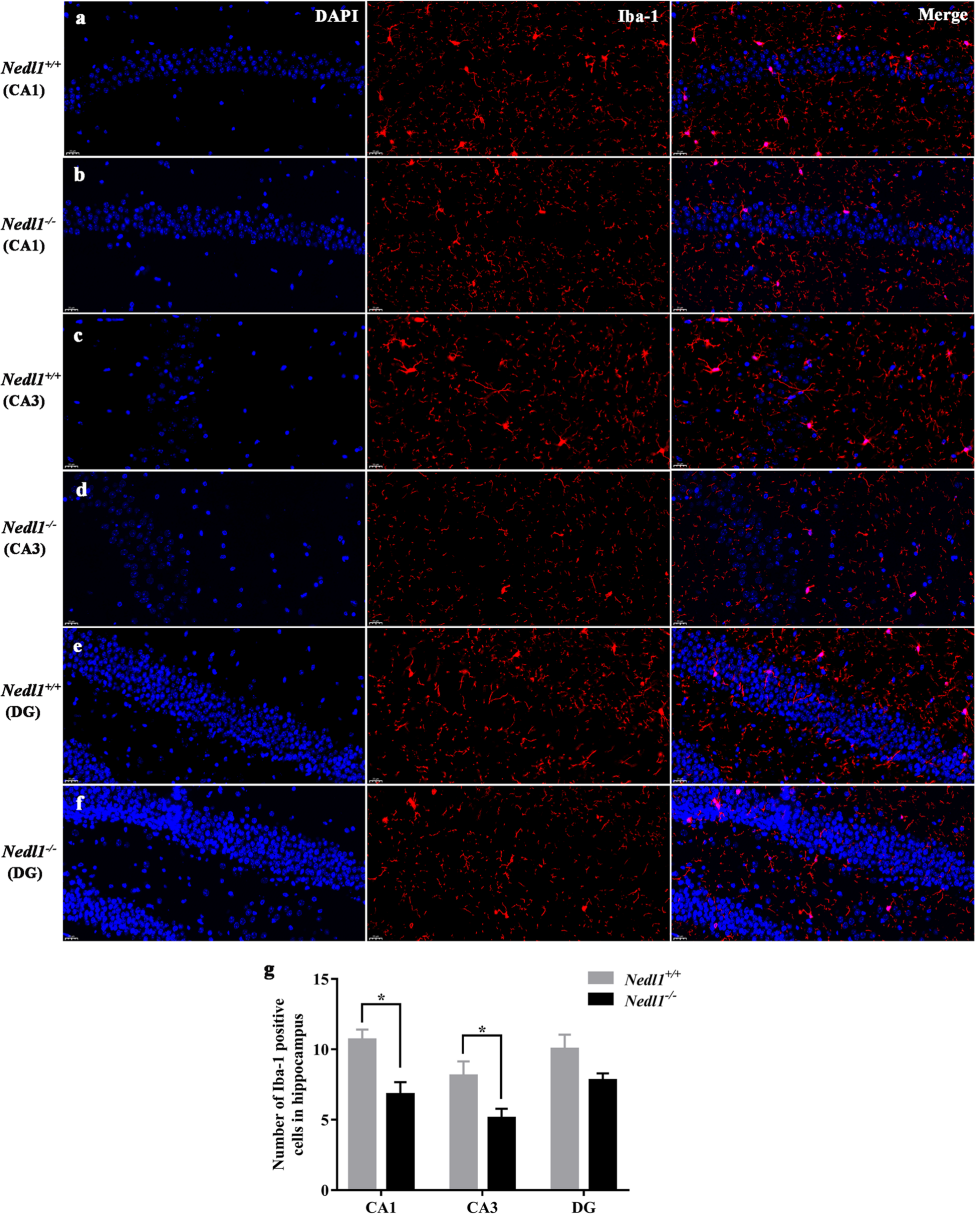
Hippocampal immunofluorescence staining of Iba-1 in the CA1, CA3 and DG regions and quantitation. *P < 0.05

H&E staining of *Nedl1*^+*/*+^ mice after epilepsy showed that the neurons were deeply stained with disordered cell arrangement; cells in the DG area were pyknotic, and the cell morphology was changed. *Nedl1* knockout reversed hippocampal neuronal damage. The numbers of neurons and astrocytes (GFAP-positive cells) in the hippocampus showed no significant difference between *Nedl1*^+*/*+^ and *Nedl1*^*−/−*^ mice (Fig. 4). In the hippocampal CA1 and CA3 regions, the number of microglia in *Nedl1*^*−/−*^ mice was significantly lower than that in *Nedl1*^+*/*+^ mice (*P* = 0.004 and 0.027, respectively). The number of microglia in the DG area did not differ between the two groups (*P* = 0.074, Fig. 5).

### DEGs are enriched in T-cell-related pathways

**Fig. 6:**
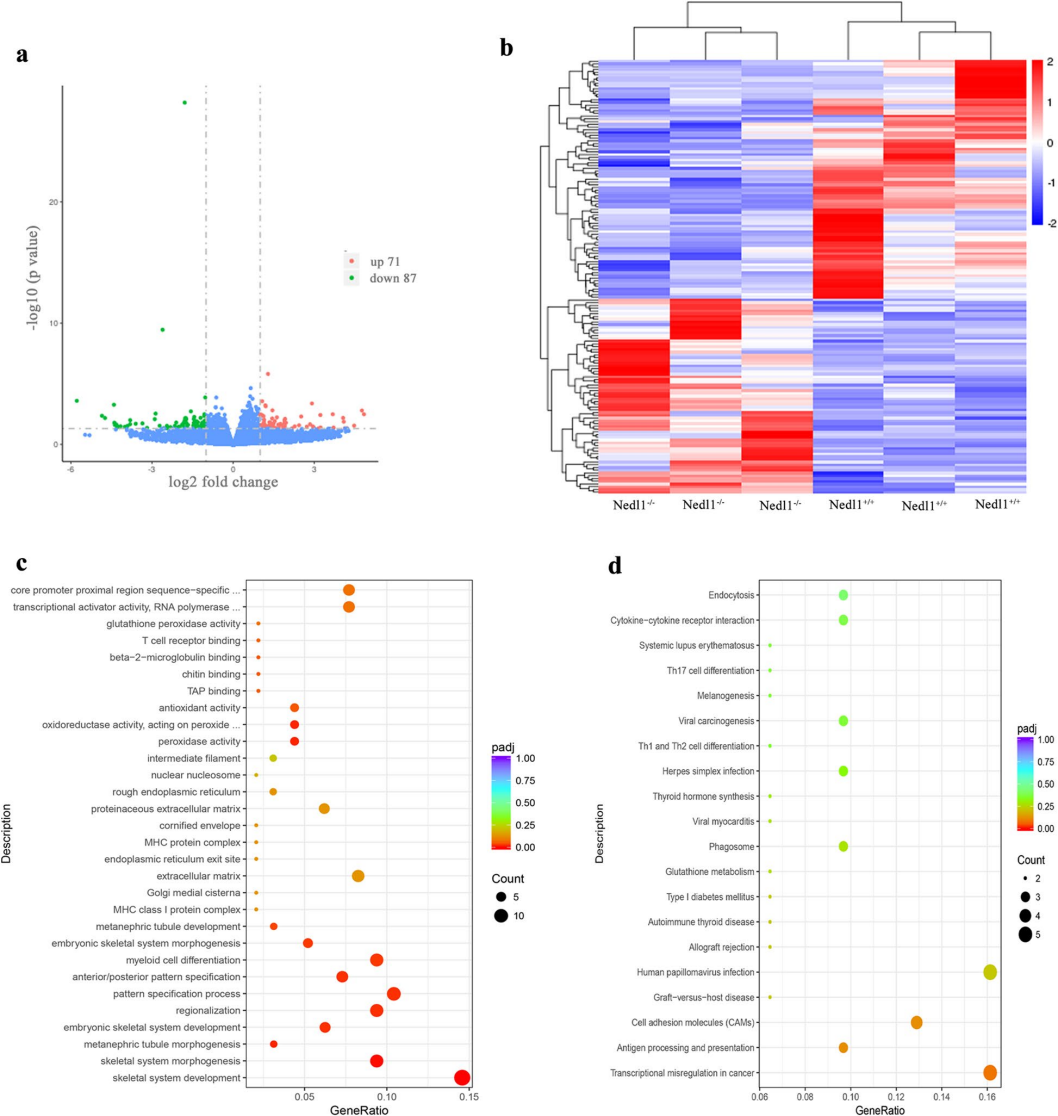
Differentially expressed genes in *Nedl1*^*−/−*^ mice compared with *Nedl1*^+*/*+^ after epilepsy. **a** The volcanic map of the differentially expressed genes. **b** The hierarchical clustering analysis and heatmap of the differentially expressed genes. Red indicates up-regulation and blue indicates down-regulation. **c** GO annotation and enrichment analysis. **d** KEGG annotation and enrichment analysis

A total of 158 DEGs were found in *Nedl1*^*−/−*^ mice compared to *Nedl1*^+*/*+^ mice, including 71 up-regulated and 87 down-regulated genes (Fig. 6a, b). The DEGs were sequenced in accordance with the fold change value. In *Nedl1* knockout mice, *Hoxb7*, *Hoxb8*, *Hoxa7*, and *Hoxc6* were at the top of the list of downregulated genes with the highest fold change values. *Gm9780* and *Gm10409* were at the top of the list of upregulated genes, but they were predicted genes. Considering the functions of genes, in the list of up-regulated genes, we focused on *H2-Q6* and *H2-Q7*.

GO functional annotation showed that the DEGs were enriched in T cell receptor binding in terms of molecular function, and the DEGs were enriched in the Histocompatibility Complex class (MHC) protein complex in terms of cellular component (Fig. 6c). KEGG pathway enrichment analysis showed that the DEGs were enriched in the T cell differentiation pathway (Fig. 6d).

## Discussion

In this study, we investigated the relationship between *Nedl1* and epilepsy by inducing epilepsy in *Nedl1*^*−/−*^ mice through pilocarpine injection. We found that *Nedl1* knockout reduced mortality and seizure severity in the pilocarpine-induced epileptic model. The patch-clamp results also confirmed this finding, showing that pilocarpine injection only induced slight changes of the electrical activity in *Nedl1*^*−/−*^ mice. Although the patch-clamp results showed that *Nedl1*^*−/−*^ mice exhibited more action potentials than *Nedl1*^+*/*+^ mice before pilocarpine incubation, we did not detect clinical seizures in *Nedl1*^*−/−*^ mice over a long period.

Microglial activation is an important process in the pathogenesis of epilepsy [14]. Microglial activation has been well described in human and experimental temporal lobe epilepsy [15, 16]. Microglial cells are immune effector cells in the central nervous system, and play an important role in the inflammatory reactions in the brain [17]. The pro-inflammatory effect of microglia is a driver of epileptogenesis. Here, we found reduced microglial activation in the hippocampus of *Nedl1*^*−/−*^ mice compared to *Nedl1*^+*/*+^ mice, which may weaken the inflammatory response and play a neuroprotective role.

A total of 158 DEGs were identified by RNA sequencing, which were enriched in T-cell-related pathways, and the expression levels of the genes *H2-Q6* and *H2-Q7* were significantly up-regulated in *Nedl1*^*−/−*^ mice. The *H2-Q7* gene, which is also known as *Qa-2*, belongs to the MHC Ib family and serves as a functional analog of HLA-G in mice [18, 19]. In humans, H2-Q7 can regulate immune responses, inhibit NK cell-mediated cytolytic effects, interfere with immune tolerance, inhibit CD4^+^ and NK cell responses, and limit antigen presentation by CD8^+^ T cells [20]. H2-Q7 has an inhibitory effect even at low expression levels [21]. Therefore, the upregulation of *H2-Q7* and other genes in *Nedl1*^*−/−*^ mice, as well as the reduced activation of microglia, could suppress immune and inflammatory responses, which may reduce seizures. However, further investigation of the relevant mechanisms is still necessary.

NEDL1, which was originally found in neurons, can enhance p53 activity [22, 23]. The increased expression of p53 was found in the hippocampus of rat models of post-traumatic epilepsy or drug-resistant epilepsy, whereas lack of p53 showed protective effects against epileptic damage [24, 25]. p53 expression is also increased in the hippocampus of patients with refractory temporal lobe epilepsy [26]. Therefore, *Nedl1* gene knockout may play a protective role in the progression of epilepsy. However, the detailed relationship between p53 and epilepsy in *Nedl1* knockout mice was not investigated in this study.

Muller et al. assessed the behavioral and cognitive changes of C57BL/6 mice after pilocarpine treatment and found that the behavioral and cognitive changes of epileptic mice could reflect some behavioral abnormalities in patients with epilepsy [27]. Therefore, animal models of pilocarpine-induced seizures have been widely used to study the symptoms of epilepsy, particularly temporal lobe epilepsy, including behavioral changes related to learning and memory [27, 28]. Based on previous reports, pilocarpine-induced epileptic mice have impaired spatial learning and memory [29]. Similar spatial learning disabilities were observed in mice after hippocampal CA1 damage [30]. In this study, results of the Barnes maze test showed that the *Nedl1*^+*/*+^ mice had impaired learning and memory after pilocarpine injection, which was consistent with literature reports. *Nedl1* knockout ameliorated the learning and memory impairment induced by epilepsy.

In the pilocarpine-induced epileptic model, neuronal damage, glial cell proliferation, and mossy fiber sprouting occur in the hippocampus [29]. Neuronal damage in the CA1 and CA3 regions of the hippocampus was observed 1 week after SE [31]. In this study, the DG area of the hippocampus was found to be severely damaged 1 month after epilepsy. The neurons showed evident pyknosis, disordered arrangement, and morphological changes. However, *Nedl1* knockout mice showed no significant changes in cell morphology in the DG region. Therefore, *Nedl1* knockout has a neuroprotective effect. GFAP is a specific marker for astrocytes, which may play a key role in epilepsy because of their impaired or dysfunctional function [32]. Chronic astrocyte proliferation can induce epilepsy [33]. However, in this study, no significant difference in the number of hippocampal astrocytes was found between *Nedl1*^*−/−*^ mice and *Nedl1*^+*/*+^ mice. Therefore, the protective effect of Nedl1 is not related to astrocyte proliferation.

This study also had several limitations. First, we only used the Barnes maze test to evaluate learning and memory. Several other methods should also be considered, such as Morris water maze, Y-maze, and radial maze tests. Second, the DEGs were not verified by q-PCR. Third, further mechanistic studies are needed.

## Conclusions

In this study, we demonstrated that *Nedl1* knockout reduced the severity of seizures, alleviated the impairment of learning and memory, reversed the damage to hippocampal neurons, and alleviated microglial activation. RNA sequencing found that the DEGs were enriched in T-cell-related pathways. These results suggest NEDL1 as a potential therapeutic target for the treatment of epilepsy.

## Supplementary Information

Supplementary Material 1.

Supplementary Material 2.

## Data Availability

The data that support the findings of this study are available from the corresponding author upon reasonable request.

## Abbreviations

DEGs: Differentially expressed genes
DG: Dentate gyrus
FPKM: Fragments per kilobase of transcript per million mapped reads
GO: Gene ontology
KEGG: Kyoto Encyclopedia of Genes and Genomes
NEDL1: NEDD4-like ubiquitin protein ligase-1
SE: Status epilepticus
