# Supplementary material for: Nedl1 knockout ameliorates cognitive impairment and improves epilepsy threshold in pilocarpine-induced epileptic mice
Source: Acta Epileptol. 2025 Jan 13;7:5. doi: 10.1186/s42494-024-00186-z (PMC11960318; doi:10.1186/s42494-024-00186-z)
Supplement: Supplementary file 2 — Supplementary Material 2. [file 42494_2024_186_MOESM2_ESM.docx]

**Additional file 2.**

Table S1. *P* values calculated using the Scheirer-Ray-Hare test.

| Index | *P* value | | |
| --- | --- | --- | --- |
|  | Group | Time | Group*Time |
| Number of errors | 0.012 | <0.001 | 0.459 |
| Latency to find the escape box | 0.632 | 0.539 | 0.344 |
